# Supplementary material for: Extra base hits: Widespread empirical support for instantaneous multiple-nucleotide changes
Source: PLoS One. 2021 Mar 12;16(3):e0248337. doi: 10.1371/journal.pone.0248337 (PMC7954308; doi:10.1371/journal.pone.0248337)
Supplement: S1 Table — E[ω]: the mean ω value for the 1H model. E[ω]:2HE[ω]:1H: the ratio of mean ω estimates from 2H and 1H models. δ:3H+δ:2H: the ratio of δ estimates from 3H+ and 2H models. The datasets are sorted by increasing values of the E[ω]:2HE[ω]:1H column. Genes where there was significant evidence (LRT p < 0.05) of non-zero 2H rates are bolded, and those where there is evidence of non-zero 3H rates is underlined. (PDF) [file pone.0248337.s001.pdf]

S1 Table. Estimated ω rate distributions for benchmark datasets for different models on the benchmark datasets. $E[\omega]$: the mean ω value for the 1H model. $\frac{E[\omega]:2H}{E[\omega]:1H}$: the ratio of mean ω estimates from 2H and 1H models. $\frac{\delta:3H+}{\delta:2H}$: the ratio of δ estimates from 3H+ and 2H models. The datasets are sorted by increasing values of the $\frac{E[\omega]:2H}{E[\omega]:1H}$ column. Genes where there was significant evidence (LRT $p < 0.05$) of non-zero 2H rates are bolded, and those where there is evidence of non-zero 3H rates is underlined.

Gene	1H				2H				3H+			
	$E[\omega]$	$\omega_1(p_1)$	$\omega_2(p_2)$	$\omega_3(p_3)$	$\frac{E[\omega]:2H}{E[\omega]:1H}$	$\omega_1(p_1)$	$\omega_2(p_2)$	$\omega_3(p_3)$	$\frac{\delta:3H+}{\delta:2H}$	$\omega_1(p_1)$	$\omega_2(p_2)$	$\omega_3(p_3)$
**β-globin**	0.28	0.0073 (32.4%)	0.28 (58.9%)	1.3 (8.66%)	0.6	0.00071 (28.4%)	0.17 (61.5%)	0.64 (10.1%)	0.86	0.0035 (30.2%)	0.19 (61.1%)	0.7 (8.68%)
**Vertebrate Rhodopsin**	0.12	0.0097 (58.8%)	0.17 (30.3%)	0.54 (10.9%)	0.7	0.0085 (60.6%)	0.12 (29.6%)	0.4 (9.79%)	0.75	0.0088 (60.6%)	0.13 (29.8%)	0.41 (9.63%)
Flavivirus NS5	0.047	0.0026 (63.9%)	0.07 (26.5%)	0.28 (9.57%)	0.73	0.0031 (67.1%)	0.062 (25.5%)	0.22 (7.4%)	0.68	0.0031 (66.8%)	0.063 (25.7%)	0.22 (7.55%)
**Drosophila *adh***	0.1	0 (50.1%)	0.1 (33.8%)	0.43 (16%)	0.73	0 (38.1%)	0.047 (42.5%)	0.29 (19.4%)	0.76	0 (37.2%)	0.046 (43.2%)	0.29 (19.6%)
**COXI**	0.07	0.0027 (75.4%)	0.033 (17.3%)	0.14 (7.33%)	0.79	0.0031 (79%)	0.035 (14%)	0.12 (6.99%)	1	0.0031 (79.3%)	0.036 (13.9%)	0.12 (6.87%)
**Sperm lysin**	1.1	0.11 (36.9%)	1.1 (41.7%)	2.9 (21.4%)	0.83	0.096 (37.2%)	1 (43.8%)	2.5 (19%)	0.87	0.1 (37.6%)	1 (43.7%)	2.4 (18.7%)
**Hepatitis D virus antigen**	0.48	0.033 (46.2%)	0.4 (32.4%)	1.6 (21.4%)	0.84	0.038 (48.7%)	0.37 (30.2%)	1.3 (21.2%)	0.9	0.037 (48.3%)	0.36 (29.9%)	1.3 (21.7%)
**Camelid VHH**	0.95	0.12 (34%)	0.73 (40.8%)	2.5 (25.1%)	0.89	0.1 (34.7%)	0.66 (40.4%)	2.2 (24.9%)	0.92	0.1 (34.4%)	0.66 (40.2%)	2.2 (25.5%)
**HIV-1 *RT***	0.19	0.016 (71.4%)	0.35 (22.2%)	1.6 (6.38%)	0.93	0.015 (70.9%)	0.31 (22.3%)	1.5 (6.82%)	0.93	0.015 (71.1%)	0.31 (22.2%)	1.5 (6.74%)
Encephalitis *env*	0.054	0.024 (77.9%)	0.028 (15.5%)	0.48 (6.62%)	0.96	0.023 (71.8%)	0.026 (21.6%)	0.46 (6.64%)	1	0.023 (75.8%)	0.026 (17.6%)	0.46 (6.64%)
**Influenza A virus HA**	0.47	0.095 (72%)	0.93 (23%)	3.7 (5.06%)	0.98	0.097 (72.8%)	0.95 (22.6%)	3.7 (4.69%)	0.99	0.098 (73.2%)	0.98 (22.4%)	3.8 (4.41%)
HIV-1 *vif*	0.84	0.14 (61.5%)	0.83 (20.6%)	3.2 (17.9%)	0.99	0.16 (66.2%)	0.96 (16.4%)	3.2 (17.4%)	0.16	0.16 (65.6%)	0.9 (16.2%)	3.2 (18.2%)
Primate Lysozyme	0.61	0.14 (0%)	0.22 (82%)	2.4 (18%)	1	0.14 (0%)	0.22 (82%)	2.4 (18%)	1	0.21 (16%)	0.22 (66%)	2.4 (18%)
