# Supplementary figures and images for: Extra base hits: Widespread empirical support for instantaneous multiple-nucleotide changes
Source: PLoS One. 2021 Mar 12;16(3):e0248337. doi: 10.1371/journal.pone.0248337 (PMC7954308; doi:10.1371/journal.pone.0248337)

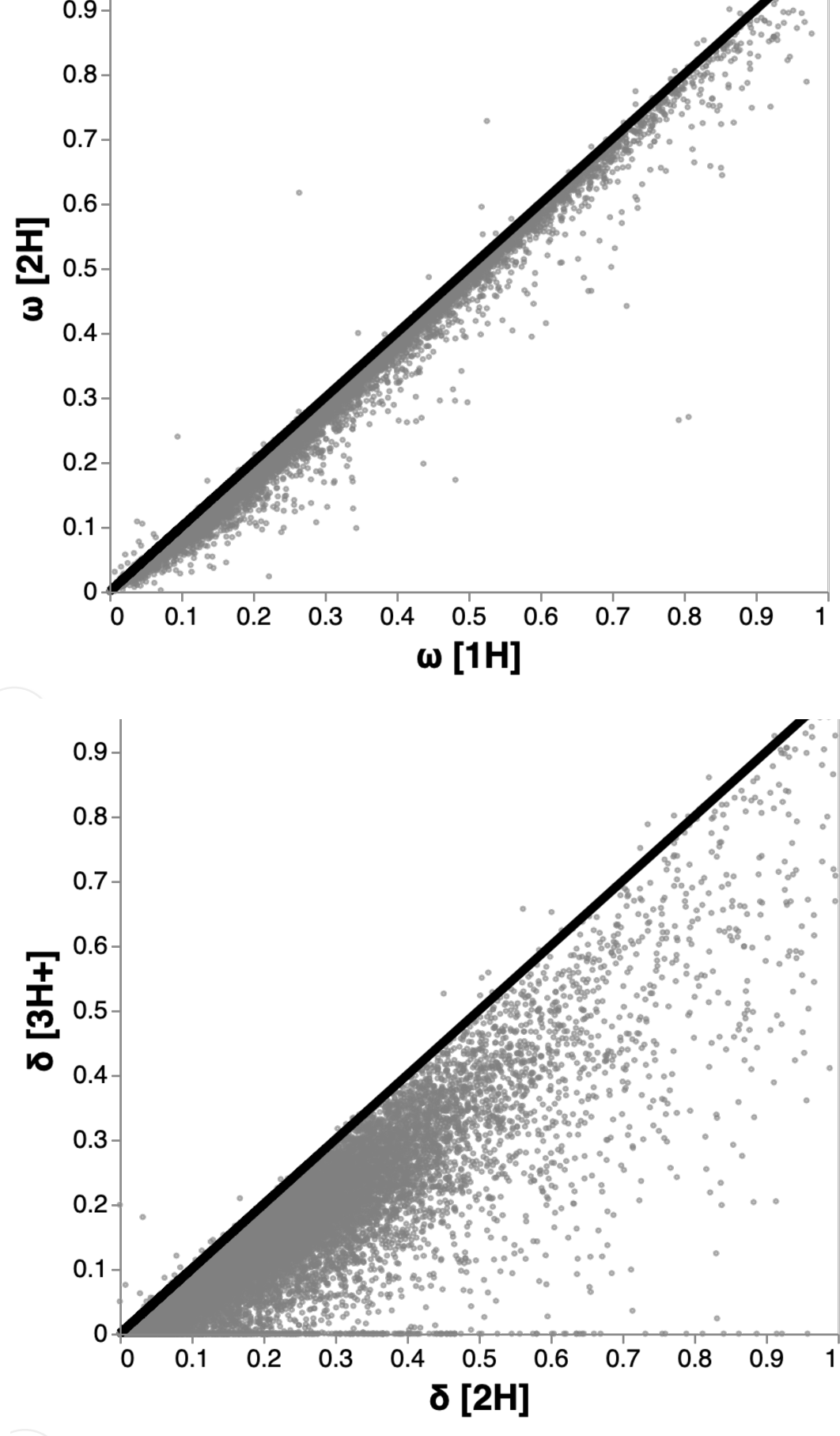

Supplement: S1 Fig — Point estimates of global rate parameters under different models for each of the empirical datasets. (TIF) [file pone.0248337.s002.tif]

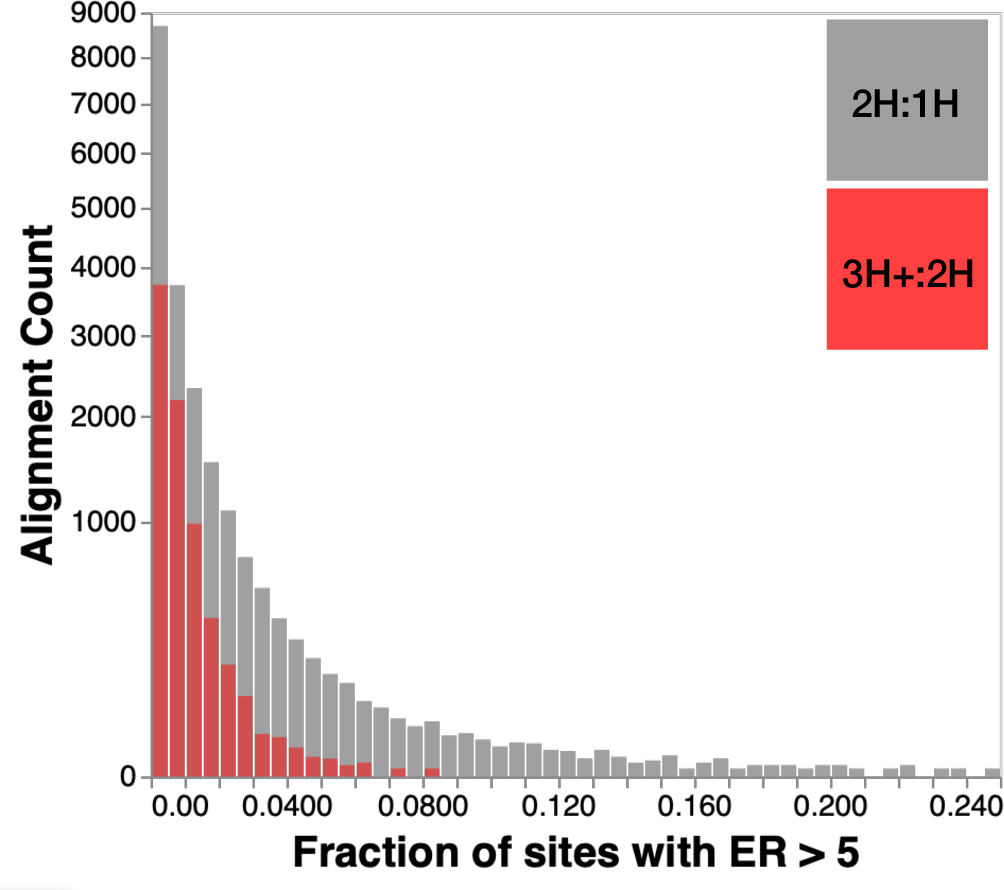

Supplement: S2 Fig — Histograms are over alignments where there was significant (p < 0.01) support for the corresponding model: 20, 338 for 2H:1H (gray) and 7664 for 3H:2H (red). (TIF) [file pone.0248337.s003.tif]

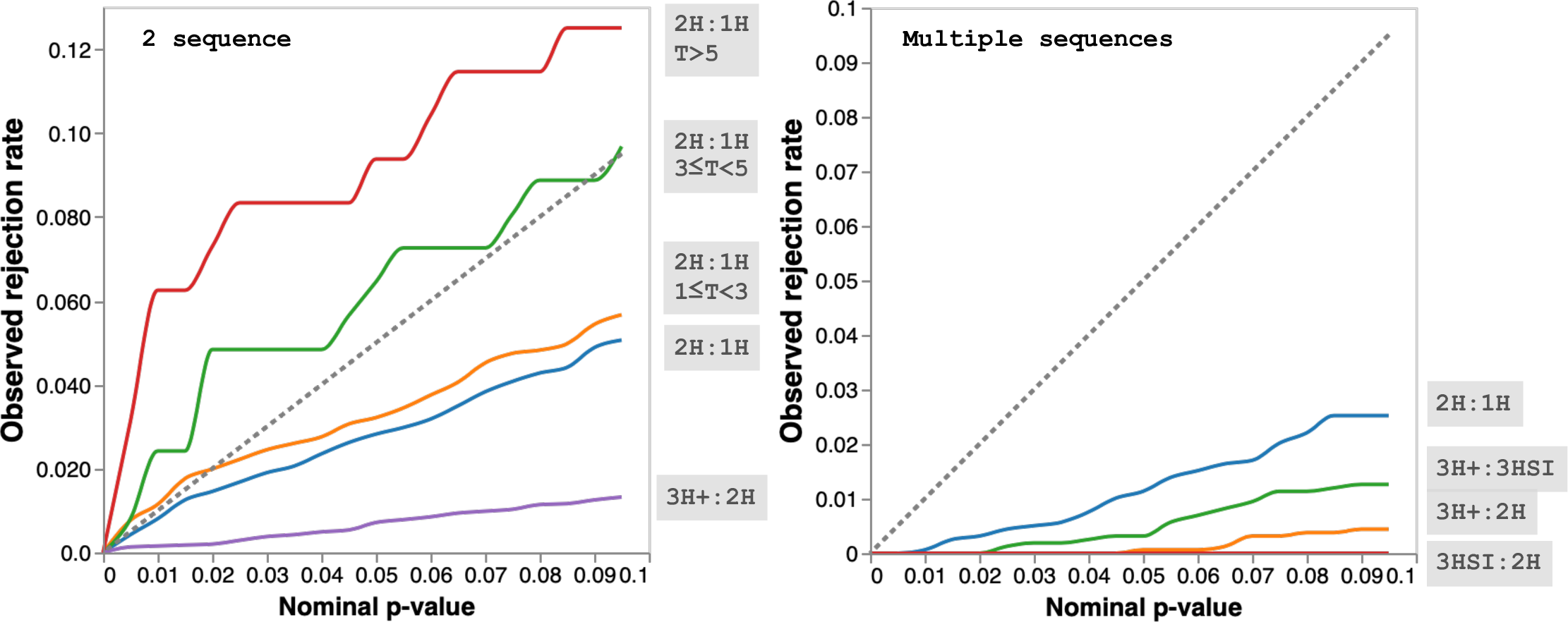

Supplement: S3 Fig — Results are shown for two sequence analyses (left) and multiple sequence analyses (right). For the two sequence simulations, we stratified the simulations by the length of the branch, T, (the range is labeled in the figure) measured in expected substitutions per site. The dotted line shows the nominal expectation (rejection rate = nominal p-value). (TIF) [file pone.0248337.s004.tif]

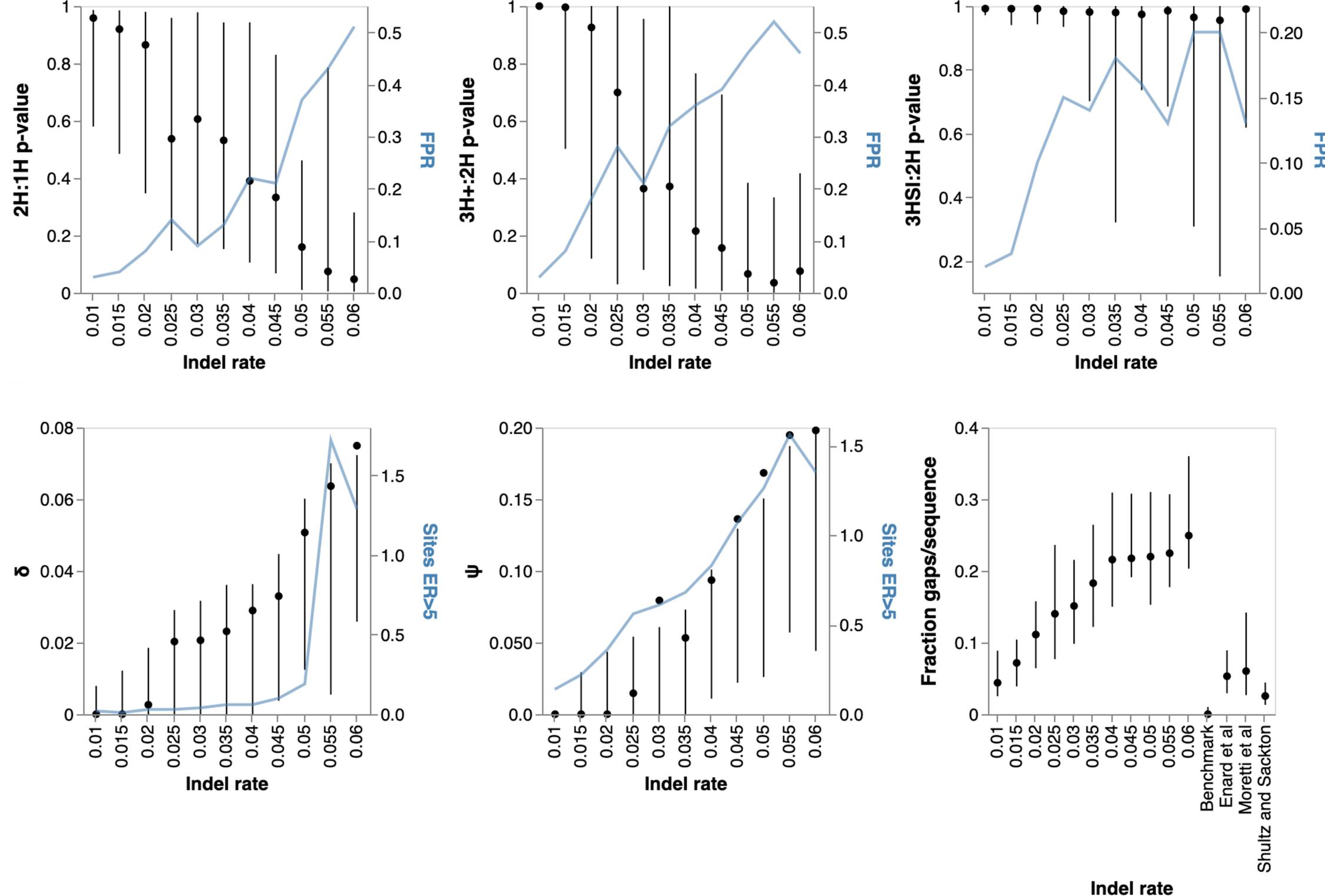

Supplement: S4 Fig — Alignments with indel were simulated using INDELible across using the Dropsophila adh tree and alignment length using GY94 M3 model with site-to-site ω variation. LRT p-values and rejection rates (FPR, at p ≤ 0.05) are shown for different tests in the top row. The bottom row shows estimated δ and ψ rates as a function of simulated indel rates, as well as the number of sites inferred to have high evidence ratios (ER) for 2H or 3H modes. The plot on the bottom right shows the average fraction of a sequence that in an alignment that is comprised of gaps is shown for simulated data, and empirical collections. (TIF) [file pone.0248337.s005.tif]

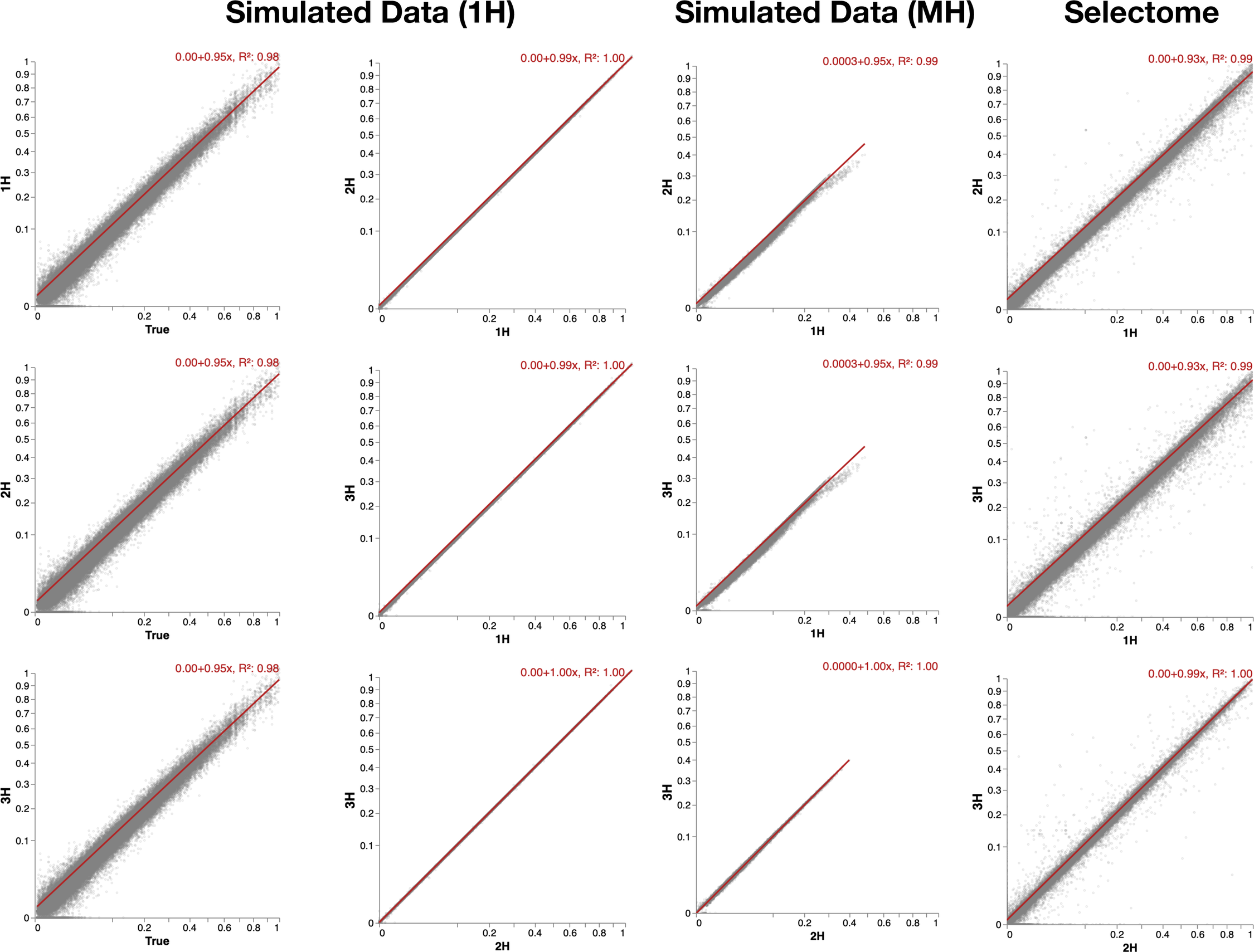

Supplement: S5 Fig — Simulated Data (1H): null simulations (1H model). Simulated Data (MH): power simulations. Selectome: empirical data. Red lines are drawn with least squares linear regression whose estimates slopes and intercepts as well as proportions of variance (R2) explained are added to each plot. (TIF) [file pone.0248337.s006.tif]
