# Supplementary material for: Extra base hits: Widespread empirical support for instantaneous multiple-nucleotide changes
Source: PLoS One. 2021 Mar 12;16(3):e0248337. doi: 10.1371/journal.pone.0248337 (PMC7954308; doi:10.1371/journal.pone.0248337)
Supplement: Attachment — Submitted filename: Response to Reviewers.pdf [file pone.0248337.s007.pdf]

Response to reviews of *Extra base hits: widespread empirical support for instantaneous multiple-nucleotide changes*

We would like to express our thanks to the associate editor, and the two anonymous reviewers for providing thoughtful and helpful critiques.

We appreciated the positive feedback and constructive suggestions made by the reviewers, and strove to address all the specific comments in the revision.

Below, we respond to the specific critiques in detail. We carefully edited the entire manuscript for clarity and grammatical correctness -- a major request from both reviewers. This included adding explanatory text, improving figure and table labeling, rearrganging passages for better flow, and additional references and details.

Reviewer 1

> The monotone splines in Figure 2 are very misleading especially given that it seems there are wildly different sample sizes across the X-axis. I do not see a clear scientific interpretation of a monotone spline in this context.

We adjusted the figure to (1) restrict plot domain to more densely sampled ares; (2) use a denser grid of "anchor points" and (3) use linear interpolation

> Fig S3 is ambiguously labeled

We revised the caption for clarity

> The double axes on Fig 4 (with what seem to be semi-outlandish ranges for the left-hand axis?) are virtually impossible to interpret reliably.

It appears the reviewer is referring to figure S4; we color-coded the axes to match the quantities being plotted. We agree that there is a lot of information packed into the figure.

> Based on my reading, the current manuscript version suffers from an overall lack-of-editing. It was not submitted in an "polished" form. I will be glad to review another version after the text has been significantly cleaned, properly organized, and figures have been designed to yield clear interpretations. Again, I believe the science here is solid, but it is not currently presented clearly at all.

We have attempted to do what the reviewer requests. However it is not immediately clear to us what major changes can be applied to the figures to improve interpretation. We addressed specific comments made by the reviewer and critically read and edited the text. We will be happy to make further modifications if these revisions remain unclear.

Reviewer 2

> According to the study, it seems that the majority of datasets and positions across an alignment do have preference for more complex models. It does make sense that more parameter-free models fit the data better, but I was not convinced that they produce better trees. Previous studies show that simpler models lead to more accurate branch-length estimates. I wonder whether these results are biased by the hypothesis testing method. I would suggest to compare the resulting trees of the simulation

> studies to the starting trees and look at the distances, e.g., branch-length distance. It could be that 3H models are better fitted but generate overestimated branch lengths whereas 1H models are more conservative and produce more accurate trees.

The reviewer raises an important point: does better fit modify downstream inferences? The point of our paper is not necessarily to improve phylogenetic inference : for that it may be that simpler models are sufficient. Our primary goal is to characterize the evolutionary process **with a given tree**; so our techniques are **downstream** of phylogenetic inference. This is not to say that MH models will or will not affect phylogenetic inference; rather that we focus on the characterization of the evolutionary process as a primary objective.

We implemented the suggestion to look at branch lengths: we added some discussion on the effect of model choice on branch lengths and added a sentence to the discussion to highlight the fact that better fit ≠ better biological inference. We find that all models considered here produce branch length estimates **that are almost in perfect agreement** -- linearly correlated with $R^2 > 0.99$, when the data are simulated **without** multiple hits. On empirical data, and data simulated with MH, standard models tend to slightly **overestimate** branch lengths on average (by a few percent), which would be expected, as a natural way for a model to accommodate occasional MH events having access only to 1H substitutions is to extend the period of time over which these substitutions will occur. Because MH are likely **rare** this effect is quite small.

In the revision, we added a Figure (S5) to show how branch length estimates compare between models, and a paragraph to the results section to provide interpretation.
