# Supplementary material for: Extra base hits: Widespread empirical support for instantaneous multiple-nucleotide changes
Source: PLoS One. 2021 Mar 12;16(3):e0248337. doi: 10.1371/journal.pone.0248337 (PMC7954308; doi:10.1371/journal.pone.0248337)
Supplement: Attachment — Submitted filename: Response to Reviewers.pdf [file pone.0248337.s008.pdf]

Response to reviewers of *Extra base hits: widespread empirical support for instantaneous multiple-nucleotide changes*

We would like to express our continued thanks to the editor, and the reviewers for providing thoughtful and helpful critiques to improve the overall quality of our manuscript.

We appreciated the positive feedback and constructive suggestions made by the reviewers, and strove to address all the specific comments in the minor revision.

Below, we respond to the specific critiques. In general, we have carefully reviewed and revised the manuscript in order to clear up spelling and grammatical errors, as requested.

> In addition to the errors noted by the reviewer, I have noticed the following: "signal due to misaligned" misses a word, probably "misaligned sites". "for one 384 branch-site tests, [20]" if there is one test it shouldn't have an 's'.

We have revised the text in this area.

> There are several English mistakes in some parts of the text - I suggest that the authors would thoroughly review the writing. Examples: "have have" in line 20; “traction” should be “attraction” in line 45; "these models *have* been unable" in line 44).

We have revised the text in this area.

We have made additional edits to the manuscript as requested.  I would be happy to make any further changes that may be required. However, I hope the manuscript is now satisfactory.
